# The Uncovered Role of Immune Cells and NK Cells in the Regulation of Bone Metastasis

**DOI:** 10.3389/fendo.2019.00145

**Published:** 2019-03-07

**Authors:** Ilaria Roato, Massimo Vitale

**Affiliations:** ^1^Center for Research and Medical Studies (CeRMS), A.O.U. Città della Salute e della Scienza di Torino, Turin, Italy; ^2^UOC Immunologia, IRCCS Ospedale Policlinico San Martino Genova, Genoa, Italy

**Keywords:** bone metastases, NK cells, cancer stem cells, dormancy, immune response

## Abstract

Bone is one of the main metastatic sites of solid tumors like breast, lung, and prostate cancer. Disseminated tumor cells (DTCs) and cancer stem cells (CSCs) represent the main target to counteract bone metastatization. These cells often localize in bone marrow (BM) at level of pre-metastatic niche: they can remain dormant for years or directly grow and create bone lesion, according to the different stimulations received in BM. The immune system in bone marrow is dampened and represents an appealing site for DTCs/CSCs. NK cells have an important role in controlling tumor progression, but their involvement in bone metastasis formation is an interesting and not fully investigated issue. Indeed, whether NK cells can interfere with CSC formation, kill them at the site of primary tumor, during circulation or in the pre-metastic niche needs to be elucidated. This review focuses on different aspects that regulate DTC/CSC life in bone and how NK cells potentially control bone metastasis formation.

## Introduction

Although many cancer patients benefit from more efficient treatments of primary tumors and become long survivors, the overall probability to develop metastases is increasing, making this aspect of the disease a key target for researchers and clinicians (1). Bone is one of the main metastatic sites for different solid tumors including breast, lung, and prostate cancer (2). Bone metastasis formation and evolution is strongly influenced by a complex cross talk occurring among tumor, immune, and bone cells (3, 4). BM, besides cell precursors, contains different types of resident or recirculating mature immune cells, including Dendritic cells (DC), macrophages, granulocytes, myeloid derived suppressor cells (MDSCs), NK cells, and different T and B lymphocyte subsets. Although some of these cells (i.e., macrophages, granulocytes, lymphocytes, and NK cells) are endowed with effector functions and directly involved in pathogen elimination, virtually all BM immune cells can produce a variety of cytokines, chemokines, or other factors possibly influencing the local tissue homeostasis. Moreover, subtypes of bone cells, such as osteoclasts (OCs), originate from immune progenitors and use “immune” receptor/ligand pairs to rule their maturation and also to govern their bone degradation activity, a process significantly involved in metastasis formation (5).

Immune cells are poorly effective in the control of metastasis formation and growth, and this is true also for bone metastases, in spite of the consistency of the immune system in the bone niches. The definition of immunotherapeutic approaches in the metastatic disease is nevertheless attractive, especially considering NK cells, a subset of powerful effectors of the innate immunity endowed with anti-tumor activity. These cells have been shown to kill pro-metastatic tumor initiating cells and, recently, also to control metastases in animal models. On the other hand, NK cell heterogeneity and the complexity of their functional interactions with the local tumor microenvironment indicate that specific studies need to be addressed to define their role in bone metastases.

## DTCs Colonize Bone Marrow and Activate the Bone Metastatic Vicious Cycle

In primary tumors, genetic, and epigenetic changes favor the switch of malignant cells to less differentiated forms through a process called epithelial-to-mesenchymal transition (EMT) (6). Cells rising from this switch can leave the primary tumor site becoming disseminated tumor cells (DTCs). DTCs can express cancer stem cell (CSC) profiles and properties such as resistance to chemotherapy and ability to home in BM for long time. Indeed, DTCs can migrate to distant organs and establish in BM at level of the premetastatic niches, which are induced by soluble factors or extra-cellular vescicles released in circulation by the primary tumor (7). The presence of DTCs in BM has clinical relevance, since it is associated to an increased risk to develop bone metastases (8–11).

In the BM, DTCs can also compete with hematopoietic stem cells (HSC) (12, 13) and establish in the niche by interacting with different elements including osteoblasts (OBs), endothelial cells, and Extracellular Matrix (ECM). OBs constitutively express CXCL12 and attract CXCR4-expressing tumor cells (14). Using mouse models, it has been shown that breast, lung, and prostate cancer cells overexpressing CXCR4 and CXCR7 increased their ability to extravasate and colonize bone (15, 16), and CXCR4 inhibition decreased bone and lung metastases (17, 18). Integrins and cadherins are other crucial factors for the interactions between DTCs and niches (16, 19). In breast cancer, the vascular-endothelial molecule-1 (VCAM-1) binds with high affinity α4β7 and α4β1 on OC precursors, leading to osteoclastogenesis, and α4 or VCAM-1 blocking antibodies effectively inhibit bone metastasis (20). Integrins can also interact with osteopontin (OPN), an ECM protein overexpressed in tumors and associated to tumor cell migration, metastases, and poor prognosis (21, 22).

Breast and prostate cancer DTCs can live in BM in a dormant state in pre-metastatic niche for years before starting to grow and to form metastases (5, 23). Indeed, the outgrowth of DTCs from dormant state, depends both on factors released by bone microenvironment, such as fibronectin, collagen I, and periostin (24), and by physical factors such as acid pH, hypoxia, high extracellular calcium concentration (25), which also cause disruption of the balanced physiological bone remodeling due to OC and OB activity (26). Remarkably, an increased OC activity generates the physical space for tumor expansion and induces the release from the bone matrix of molecules that further stimulate tumor cell proliferation, creating the vicious cycle (27, 28). Tumor cells in turn secrete PTHrP, activated vitamin D, tumor necrosis factor (TNF), matrix metalloproteinases (MMPs), interleukin-6 (IL-6), and other factors, which stimulate the expression of the receptor activator of nuclear factor NF-kB ligand (RANKL) on OBs, leading to the final stimulation of osteoclastogenesis from local OC precursors (3, 20).

## Interaction Between Immune System and Bone FAVOR Tumor Cell Survival and Proliferation

A fundamental molecular link between immune system and bone is represented by the axis comprising RANKL, its receptor RANK, and the natural decoy receptor osteoprotegerin (OPG) (29, 30). RANK/RANKL interaction activates osteoclastogenesis, while OPG counteracts this effect by competing with RANK to bind RANKL (31). OBs and BM stromal cells are the main producers of both RANKL and OPG in physiological conditions (32), however, B or activated T cells can influence the RANKL/OPG ratio, end eventually osteoclastogenesis by producing OPG or RANKL, respectively (33). Literature data report that T cells could directly carry on a modulatory action on OCs through production of different factors such as IL-7, RANKL, TNFα (34–38). Circulating OC precursors from bone metastatic patients have been shown to differentiate into mature OCs in a T cell dependent way, in the absence of the classical OC inducers M-CSF, and RANKL (39). On the other hand, in mouse models, it has been shown that T cells exert a fundamental anti-tumor effect, regardless of OC status. Indeed, PLCγ2-KO mice, with dysfunctional OCs and impaired T-cell activation, showed increased bone tumor growth despite protection from bone loss, whereas Lyn-KO mice with numerous OCs and increased T-cell responses, showed impaired tumor growth in bone despite enhanced OC activity and osteolysis. The injection of antigen-specific wild-type cytotoxic CD8(+) T cells in both these mouse models normalized tumor growth in bone, suggesting their important role in the regulation of tumor bone metastases (40). T cells can limit tumor cell diffusion by releasing IFNγ, which also affects osteoclastogenesis, indeed lack of IFNγ has been related to the increase of bone metastases (41).

Tumor cells modify the surrounding microenvironment, indeed it has been shown that BM from breast cancer patients differed from that of healthy subjects in its cellular composition as well as the activation status of cells from the innate immune system (macrophages, NK cells) and from the adaptive immune system (T cell subsets) (42). Many immature and suppressor immune cell types are present in bone, such as T regulatory cells, which must maintain a balanced immune-reactivity (43), and MDSCs, which stimulate osteoclastogenesis (44). In breast cancer, infiltrating T regulatory cells produce RANKL, promoting OC differentiation, activity, and subsequent bone lesions (45).

MDSCs are increased in cancer patients from 2 up to 25% (46) suppressing innate and adaptive immune response, thus sustaining tumor growth and metastatization (47). In breast cancer, MDSCs, derived from bone metastatic microenvironment, can differentiate into mature and functional OCs *in vitro* (48).

## NK Cells are Endowed With Powerful Anti-Tumor Functions

NK cells can kill a variety of tumor cells of different origin and types (49–52). This wide range of reactivity is ensured by the expression at the cell surface of several receptors capable of activating or inhibiting the main functions of NK cells, including the release of cytolytic granules (49, 53). Thus, thanks to their HLA-I-specific inhibitory receptors and a complex and heterogeneous group of activating receptors, NK cells can sense the HLA-I expression decrease that often characterizes tumor cells and recognize different ligands that can be variably induced on cells undergoing tumor transformation (Table 1). Different patterns of NK receptors are engaged during contact with pathological or non-pathological cells, regulating the activation, and the intensity of the cytolytic response (49, 50, 53, 54). Most NK cells express the FcγIII-receptor (CD16), which is a strong activator of cytotoxicity and enables NK cells to mediate the Antibody-Dependent Cellular Cytotoxicity (ADCC).

**Table 1:** Overview of the major NK cell receptors and Ligands involved in tumor cell recognition.

	**NK Receptor**	**Ligand(s)**	**Ligand expression on tumor cells**	**References**
Inhibitory receptors	KIRs^*^	HLA-I (HLA-A,B,C)	Down-regulated in certain tumor cells	(50, 54)
	CD94:NKG2A	HLA-E (non-classical HLA-I)	Down-regulated in certain tumor cells	(50, 54, 55)
	LILRB1	HLA-I (HLA-A,B,C)	Down-regulated in certain tumor cells	(50, 54)
		HLA-G (non-classical HLA-I)	Up-regulated in certain tumors	(55–57)
Activating receptors	NKp46	HSPG	Up-regulated/modified in different tumor cells	(58, 59)
		Complement Factor P (properdin)	?	(60)
		Additional still unknown ligands^**^		(50, 61)
	NKp44	HSPG	Up-regulated/modified in different tumor cells	(58, 59)
		MLL5 isoform	Ectopically expressed at the cell surface of tumor cells of hematologic and solid tumors	(62)
		PDGF-DD	Soluble factor released by several tumors (induces NKp44-dependent cytokine release)	(63)
		Nidogen-1	Decoy extracellular ligand expressed by different tumor cell lines (inhibits NKp44-dependent cytokine release)	(64)
	NKp30	HSPG	Up-regulated/modified in different tumor cells	(58, 59)
		BAT3	Up-regulated in different tumor cells (released in exosomes)	(65)
		B7-H6	Highly expressed in different tumor cells	(66)
	NKG2D	MICA/B, ULBP1-6	Up-regulated in tumors of epithelial and non-epithelial origins	(67)
	DNAM-1	CD155, CD112	Up-regulated in many tumor cell types	(68)

* *KIRs, Killer-cell immunoglobulin-like receptor; NKG2A, Natural Killer Group 2 A; LILRB1, Leukocyte Immunoglobulin Like Receptor B1; NKG2D, Natural Killer Group 2 D; DNAM-1, DNAX Accessory Molecule-1; HLA, Human Leukocyte Antigen; HSPG, Heparan Sulfate Proteoglycans; MLL5, mixed-lineage leukemia protein-5; PDGF-DD, platelet-derived growth factor—isoform dimer DD; BAT3, human leukocyte antigen (HLA)-B-associated transcript 3; MIC, MHC class I chain-related protein; ULBP, UL16 binding proteins*.

** *Different tumor cell lines bind recombinant soluble NKp46 receptors and/or are killed by NK cells in a NKp46-dependent way but the putative ligand on these cells has not yet been identified*.

NK cells can attack tumor cells by releasing pro-apoptotic factors, including TNF-α and Tumor necrosis factor-related apoptosis-inducing ligand (TRAIL) (69, 70), or cytokines capable of inhibiting tumor cell proliferation and promoting the inflammatory response, such as IFN-γ. In addition, NK cells can release chemokines (CCL3, CCL4, CCL5, and XCL1) capable of attracting T cells, DC, and monocytes (71, 72) and give rise to specific cross-talks promoting and regulating the adaptive anti-tumor response (73–75). Finally, NK cells can also amplify their recruitment at the tumor site by releasing a chemotactic form of HMGB1 molecule upon interaction with tumor cells (76).

In order to appropriately evaluate the role of NK cells in the control of tumors it should be also considered that the NK cell population is rather heterogeneous as it includes different cell subsets, each characterized by peculiar functional capabilities (77). In humans, the CD56brightCD16dim/neg (CD56^bright^) and the CD56dim/CD16bright (CD56^dim^) cells represent the two most studied NK cell types. The CD56^bright^ NK cells largely produce IFN-γ in response to monokines but are poorly cytotoxic. These cells constitute 5–10% of circulating NK cells, and, in line with their pattern of chemokine and homing receptors (i.e., CD62L, CCR7, CXCR3, and CXCR4), represent most LN-NK cells and an important fraction of tissue NK cells in different organs. The CD56^dim^ cells release IFN-γ upon triggering of major activating receptors (NKp46, NKp30, NKp44, and CD16) and are highly cytotoxic. They represent 90–95% of PB NK cells and predominate in spleen, lungs, and kidney although in different percentages. Moreover, CD56dim NK cells express chemokine receptors (CXCR1, CX_3_CR1, and CXCR4) that allow their possible recruitment to inflamed peripheral tissues (77, 78). The assessment of NK cells in tissues and the definition of their anti-tumor potential are rather complicated. Indeed, tissues comprise both potentially cytotoxic NK cells that recirculate from PB, but also stably resident cells expressing specific markers of tissue retention (CD69, CD49a, and CD103) and chemokine receptors (CCR5, CXCR6) (79–82). These latter cells may display unique functions, possibly organ-specific, not necessarily oriented to tumor cell killing.

## Role of NK Cells in the Control of Solid Tumors and Metastatic Spread

Several studies using different mice models have documented that NK cells can control tumor insurgence, growth, and metastasis dissemination (83–86). Remarkably, the role of NK cells in the control of tumors has also been suggested in different human studies. In a 11-year follow-up study on more than 8,000 healthy individuals, Imai et al. initially showed that insurgence of tumors of different types inversely correlated with the levels of natural cytotoxic activity of peripheral blood lymphocytes (87). More recently, different groups have found correlations between the quantity and the quality of tumor infiltrating or PB-NK cells and a more favorable prognosis or the lower number of metastases at diagnosis (88). In this last decade, it has also become evident that a plethora of mechanisms of tumor escape can strongly reduce the efficacy of NK cells. Within the tumor microenvironment, different immune suppressor cells (including Tregs and MDSCs), tumor-associated fibroblasts (TAF), and tumor cells can produce soluble factors (TGF-β, PGE2, IDO-derived kynurenine) which inhibit expression and function of the major activating receptors (89, 90). Similar effects on activating receptors are induced also by soluble decoy ligands shed by tumor cells or released as extracellular molecules (64, 89, 91, 92). Finally, exposure to hypoxia, which often characterizes tumor tissues, can also cause activating receptor down-regulation (93). Remarkably, some of these suppressive mechanisms, such as those induced by hypoxia and TAFs, appear to minimally affect the ADCC function (94). On the other hand, hypoxia and tumor cells can modulate the repertoire of chemokine receptors on NK cells and favor the preferential recruitment of CD56bright cells (poorly cytotoxic and unable to mediate ADCC) (95). The NK-cell recruitment into neoplastic tissues may also be influenced by the chemokine profile induced in the tumor microenvironment. Human lung and breast tumors have been shown to express higher CCL19 (a CD56bright cell attracting chemokine) and lower CXCL12 compared to their normal tissue counterpart (96), while in mice, BM with MM showed increased CXCL9 and CXCL10, decreased CXCL12, down-modulation of CXCR3 on NK cells, and selective reduction of KLRG1^−^ cytotoxic NK cells (97). Collectively, the above-described mechanisms can account for the observation that in different tumor tissues the NK cell infiltrate is often limited or constituted by CD56bright or altered (poorly functional) CD56^dim^ cells (50, 89).

Another important issue regards the so-called immune-checkpoints. Different pairs of receptor-ligands are available to the immune system to regulate or terminate excessive (dangerous) responses. Some of these receptors, such as PD-1, TIM-3, TIGIT, and SIGIRR, can be also expressed by NK cells, especially by those associated to tumors, and control different NK cell functions including cytotoxicity. Blocking or overcoming these checkpoints, by specific monoclonal antibodies or activating cytokines can improve the NK-mediated control of carcinogenesis or metastasis formation (98–101).

## The Ambiguous Role of the NK:CSC Cross-Talk in the Control of Metastasis Formation

Whether NK cells can interfere with CSC formation, or kill CSC at the site of primary tumor, during circulation, or in the pre-metastatic niches represents an interesting and still incompletely investigated issue. Several reports have indicated that NK cells can kill tumor cells with features of CSC derived from different tumors (glioma, melanoma, colon, prostate, and breast) (102). Consistent with these findings, CSCs of different origins have been shown to express or even up-regulate the ligands for NKG2D, DNAM1, and NKp30 NK-activating receptors and cells undergoing EMT showed up-regulated NKG2D-Ls (88, 102). In addition, EMT induction in lung cancer cells could promote increased NK cell-mediated metastasis-specific immunosurveillance in RAG1^−/−^ mice (103). On the other hand, it has also been shown that NK cells could induce melanoma cells to undergo EMT, upregulate the expression of stemness markers, reduce proliferative capability, thus acquiring characteristics reminiscent of the CSC phenotype. Moreover, EMT increased ability of melanoma cells to suppress NK cell cytotoxicity against tumor cells (104).

## Is there any Role for NK Cells in Bone Metastases?

Although the role of NK cells in contrasting bone metastases has been recently suggested in breast cancer preclinical models (105), an established knowledge on this issue is still lacking. BM is where NK cells mature and differentiate from CD34+ progenitors; therefore, it contains precursors at different stages. Once NK cells have matured, changes in the expression of the key receptors CXCR4 and S1P5 (down- and up-regulated, respectively) drive their egress from the CXCL12-containing BM and their recruitment to blood where the S1P5-ligand S1P is abundant (106). Besides the immature NK cell precursors, BM also contains a reservoir of mature NK cells, recirculating from the blood, which can be mobilized upon inflammatory stimuli (107). BM also includes a substantial population of resident CXCR6+CD69+ NK (BMrNK) cells, which may be poorly effective against tumor cells. Indeed, compared to classical NK cells, BMrNK cells display lower proliferative capacity, cytolytic granule content, DNAM1, and higher TIGIT expression (108).

**Figure 1:**
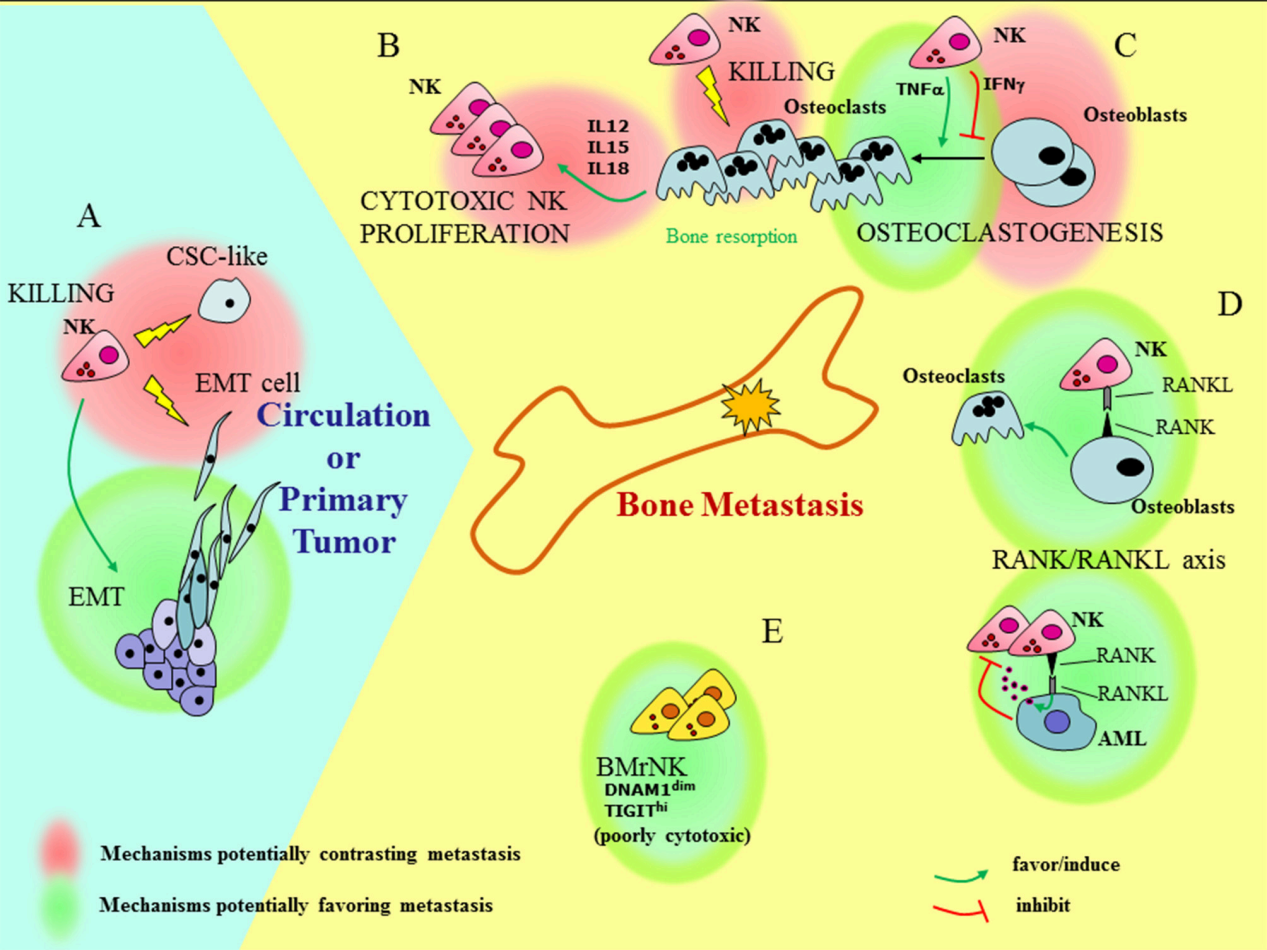
Understanding how NK cells can influence bone metastasis. Outside and inside the bone (light blue and yellow fields, respectively), different functional interactions involving BM resident, recirculating, or PB NK cells can have contrasting effects on bone metastasis formation and tumor progression. **(A)** At the site of primary tumors NK cells may favor EMT. On the other hand, NK cells can kill pro-metastatic tumor cells, such as cells that have undergone EMT (EMT cells), and cells with features of CSC (CSC-like). **(B)** OCs can induce proliferation of highly cytotoxic NK cells via the release of IL12, IL15, and IL18. **(C)** The interaction of NK cells with OCs gives rise to contrasting effects: NK cells can either favor or inhibit generation of OCs depending on the release of TNF-α or IFN-γ, respectively and kill OCs. **(D)** The RANK/RANKL axis may play a role in the cross-talk between NK cells and bone microenvironment: the signaling of RANK-L in leukemia cells (AML) can induce the release of NK-suppressing factors; on the other hand, RANKL-expressing NK cells provide signals for OC generation. **(E)** Besides recirculating PB-NK cells, BM also contains resident poorly cytotoxic NK cells (BMrNK) which may hardly eliminate tumor cells.

The heterogeneity of the NK cells in the BM and their still poorly defined interaction with the metastatic niche, together with the possible cross-talk between PB-NK and CSC/EMT cells add a layer of complexity to the issue of how NK cells can influence bone metastasis formation and progression (Figure 1). In the bone, the RANK-RANK-L axis, whose deregulation is important in metastasis formation, also influences NK cells. The signaling of RANK-L in leukemia cells can induce the release of NK-suppressing factors (109), whereas, under inflammatory conditions (knee arthritis) NK cells can stimulate OCs through activation of RANKL pathway (110). NK cells can either favor or inhibit generation of OCs depending on the release of TNF-α or IFN-γ, respectively (111, 112). On the other hand, OCs have been shown to contribute to the induction of efficient NK cells, capable of inhibiting growth of poorly differentiated tumors in humanized BLT mice (113). This effect is in line with the ability of OCs to produce NK-stimulating cytokines such as IL-12, IL-15, and IL-18. Finally, OCs are targets of NK cells, as they express MHC class I molecules at low levels and are killed by IL-2 treated NK cells (114).

## Concluding Remarks

Understanding the reason why and how in many patients' metastases can overcome the surveillance of NK cells is still poorly understood. Studies are rapidly progressing to define how to properly activate NK cells by cytokine combinations and unleash their potential by blocking their checkpoint receptors. The crucial mechanisms that govern entrance and egress of NK cells in the bone metastatic niche and modulate the NK cell killing capability within the bone lesions are lacking. Addressing these questions will significantly increase the therapeutic options for NK cells in the treatment of bone metastatic disease.

## Author Contributions

IR revised the literature and wrote the paragraphs concerning bone metastasis. MV revised the literature and wrote the paragraphs concerning NK cells.

### Conflict of Interest Statement

IR was supported by Roche Foundation. The funder played no role in the study design, the collection, analysis or interpretation of data, the writing of this paper or the decision to submit it for publication. The remaining author declares that the research was conducted in the absence of any commercial or financial relationships that could be construed as a potential conflict of interest.
